# Intercellular signal transduction within the mother cell compartment during *Bacillus subtilis* sporulation

**DOI:** 10.1093/femsle/fnag055

**Published:** 2026-05-05

**Authors:** Nobuki Kuwabara, Masato Anzue, Shin-ichi Miyoshi, Tsutomu Sato, Daisuke Imamura

**Affiliations:** Department of Frontier Bioscience, Hosei University, Koganei, Tokyo 184-8584, Japan; Department of Frontier Bioscience, Hosei University, Koganei, Tokyo 184-8584, Japan; Research Center for Intestinal Health Science, Okayama University, Okayama 700-8530, Japan; Department of Frontier Bioscience, Hosei University, Koganei, Tokyo 184-8584, Japan; Department of Frontier Bioscience, Hosei University, Koganei, Tokyo 184-8584, Japan; Research Center for Intestinal Health Science, Okayama University, Okayama 700-8530, Japan

**Keywords:** *Bacillus subtilis*, sporulation, sigma cascade, intercellular signal transduction

## Abstract

Intercellular signaling contributes to the spatiotemporal regulation of gene expression during sporulation in *Bacillus subtilis*. The mother cell transcription factor σ^E^ is initially produced as an inactive precursor protein pro-σ^E^ and activated by the processing enzyme SpoIIGA in response to the forespore-produced putative signaling molecule SpoIIR. However, the mechanism underlying the SpoIIR-mediated signal transduction remains poorly understood. In this study, we showed that the *spoIIR*-positive, *spoIIGA*-deleted strain was able to induce SpoIIGA-dependent pro-σ^E^ processing in co-cultured *spoIIR*-deleted, *spoIIGA*-positive strains. This signaling was dependent on SpoIIR expression and did not involve DNA transfer. Extracellular materials including secreted proteins and membrane vesicles were unlikely to be involved in this signaling pathway. Interestingly, cessation of co-incubation shaking enhanced the signaling, while the addition of membrane-solubilizing detergent abolished it. In addition, SpoIIR signaling did not necessitate release from the forespore membrane or extracellular translocation. A SpoIIR variant lacking the putative signal peptide-like hydrophobic domain produced solely in the mother cell compartment was still able to activate pro-σ^E^. Overall, the study findings suggested that the forespore-produced SpoIIR is neither secreted nor externally translocated. Instead, SpoIIR appeared to be transferred into the mother cell compartment and interacts with the SpoIIGA cytoplasmic domain to trigger pro-σ^E^ processing.

## Teaser

SpoIIR was able to activate SpoIIGA within the mother cell compartment in contrast to the previously expected signal transduction at extracellular domain of SpoIIGA.

## Introduction

The mechanisms underlying intercellular communication during cell differentiation remain a fundamental question in developmental and cell biology. In the Gram-positive bacterium *Bacillus subtilis*, intercellular signal transduction plays a critical role in coordinating the gene expression of two cell types during spore formation (Errington 1993). Sporulation is initiated in response to nutrient deprivation. The initial morphological alteration is the formation of an asymmetrically positioned septum, which divides the cell into the larger mother cell and the smaller forespore. Asymmetric division triggers the activation of the first compartment-specific RNA polymerase σ factor (σ^F^) in the forespore (Stragier and Losick 1996). σ^F^ induces the expression of about 48 genes in the forespore, including *spoIIR*, whose product signals the activation of σ^E^ in the mother cell (Wang et al. 2006). σ^E^ then triggers 262 genes in the mother cell (Eichenberger et al. 2004). The σ^F^- and σ^E^-regulated compartment-specific gene expression in the forespore and mother cell, respectively, induces further morphological alterations. The mother cell membrane migrates around the forespore, known as engulfment, generating the forespore within the mother cell. After completing engulfment, the late forespore-specific transcription factor (σ^G^) becomes active and displaces σ^F^ in the forespore. σ^G^-produced signaling proteins in the forespore trigger σ^K^ activation in the mother cell to direct the transcription of late mother cell-specific genes (Kroos 2007). The spatiotemporally regulated expression of sporulation genes by four compartment-specific sigma factors plays a central role in the developmental process. Ultimately, it induces spore maturation and its release upon mother cell lysis.

**Figure 1:**
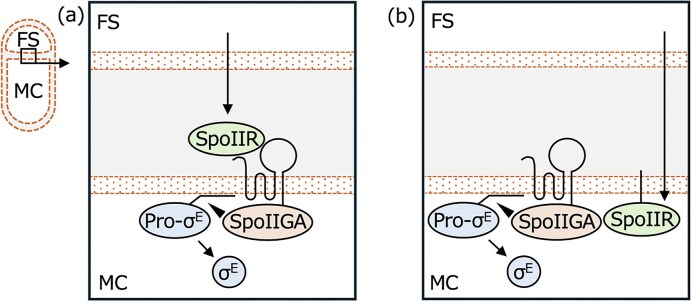
Schematic representation of SpoIIR signaling from the forespore to the mother cell. *B. subtilis* cells are divided into a larger mother cell (MC) and a smaller forespore (FS) during sporulation (left). a; Previously proposed pathway. SpoIIR produced in the forespore is secreted and interacts with the extracellular domains of SpoIIGA to activate SpoIIGA-dependent pro-σ^E^ processing in the mother cell. b; Newly proposed pathway. SpoIIR produced in the forespore is neither secreted nor externally translocated. SpoIIR is transmitted into the mother cell compartment and interacts with the cytoplasmic domain of SpoIIGA to activate SpoIIGA-dependent pro-σ^E^ processing.

σ^E^ is encoded by the *sigE* (*spoIIGB*) gene as an inactive precursor protein pro-σ^E^ and activated by cleavage of its N-terminal 27 amino acid extension (LaBell et al. 1987, Miyao et al. 1993). *sigE* is cotranscribed in an operon with *spoIIGA* (Kenney and Moran Jr 1987), whose product has been reported to be an aspartic protease that cleaves the pro-sequence of pro-σ^E^ (Imamura et al. 2008, Imamura et al. 2011). SpoIIR, produced in the forespore in a manner dependent on σ^F^, is required for pro-σ^E^ processing in the mother cell (Zhang et al. 1996). SpoIIT, implicated in the signaling pathway, acts as a co-activator of SpoIIGA-dependent pro-σ^E^ processing (Meeske et al. 2016). However, SpoIIR and SpoIIGA were necessary and sufficient for reconstituting accurate and abundant pro-σ^E^ processing in the heterologous host *Escherichia coli* (Imamura et al. 2008). Culture supernatant concentrated 3- to 10-fold by ultrafiltration from *B. subtilis* cells engineered to produce σ^F^ during growth, in which its regulon including SpoIIR is presumably produced, was capable of stimulating pro-σ^E^ processing in protoplasts of *B. subtilis* cells that had been engineered to express SpoIIGA and pro-σ^E^ (Hofmeister et al. 1995). Thus, SpoIIR is recognized as the putative signaling molecule involved in the pro-σ^E^ processing pathway from the forespore to the mother cell and is believed to be released from the forespore and transported to the region between the forespore and the mother cell membranes. Consistent with this hypothesis, SpoIIR has an N-terminal signal peptide-like hydrophobic domain (Karow et al. 1995). SpoIIGA is embedded in the mother cell membrane with N-terminal 5 transmembrane domains, and a pull-down assay revealed its interaction with SpoIIR (Imamura et al. 2008). These results suggest that the forespore-synthesized SpoIIR is secreted into the space between the forespore and mother cell membranes and interacts with the extracellular domains of SpoIIGA to trigger pro-σ^E^ processing (see Fig. 1a). Three extracellular domains of SpoIIGA—the N-terminal 6 or 7 residues, an extremely short loop of 1 or 2 residues between transmembrane domains 2 and 3, and a large loop between transmembrane domains 4 and 5—have been predicted (Londono-Vallejo 1997a). However, the entire large extracellular loop is dispensable for SpoIIGA activity. The substitution of conserved residues in the N-terminal end of SpoIIGA revealed that only the D6A substitution exhibited a noticeable effect, whereas substitutions such as D6E and other conserved residues did not impact SpoIIGA function. Hence, the mechanism by which SpoIIR transmits the signal from the forespore to SpoIIGA, embedded in the mother cell membrane, requires elucidation.

Although SpoIIR has N-terminal signal peptide-like hydrophobic domain, it is unclear whether SpoIIR is secreted. Anti-FLAG antibody-based Western blot analysis in the *E. coli* reconstitution system of pro-σ^E^ processing showed that SpoIIR-FLAG2 appeared as a single band within the cell envelope fraction but not in the soluble fraction and the products, whose size correspond to the SpoIIR without putative signal peptide, was not observed (Imamura et al. 2008). SpoIIR-His6, expressed in *B. subtilis* under the control of an IPTG-inducible hyper spac promoter, was observed as the full-length protein (25.1 kDa) but not in its secreted cleaved size (23.7 kDa) whereas an abnormally small product (17 kDa) was detected (Diez et al. 2012). Londoño-Vallejo engineered *B. subtilis* to express a chimeric protein, fusing the N-terminal signal peptide-like domain of SpoIIR to the soluble domain of levansucrase, under the *spoIIAC561* genetic background, which produced a modified form of σ^F^ resulting in a higher expression of *spoIIR* (Illing and Errington 1991). The chimeric protein was visible at a size corresponding to the fusion protein, and smaller products were not detected (Londoño-Vallejo 1997a). Furthermore, the signal peptide-like domain of SpoIIR was replaced by the conventional N-terminal membrane domain (MD) of SpoIIQ, in which MD_SpoIIQ_-SpoIIR is expected to be translocated to outside the forespore but not released from the membrane (Londoño-Vallejo et al. 1997b). This version of the MD_SpoIIQ_-SpoIIR fully complemented the sporulation defect of the Δ*spoIIR* strain, indicating that SpoIIR release from the forespore membrane is not required for the signaling (Londoño-Vallejo 1997a). Therefore, whether SpoIIR is secreted remains unclear.

*Bacillus subtilis* cells treated with cerulenin, a specific inhibitor of fatty acid biosynthesis, at the onset of sporulation allowed formation of the asymmetric septum and σ^F^ activation, but pro-σ^E^ processing was impaired (Schujman et al. 1998), suggesting that signal transduction by SpoIIR requires *de novo* fatty acid biosynthesis. Diez et al. (2012) demonstrated that fatty acid synthesis is required in the forespore (i.e. the donor of the SpoIIR signal). These observations suggest the potential involvement of *de novo* fatty acid synthesis in SpoIIR transfer from the forespore to the mother cell.

The formation of tubular membranous structures has been reported in several bacterial species including nanotubes in *B. subtilis* and *E. coli* (Dubey and Ben-Yehuda 2011, Pande et al. 2015), as well as other bacteria producing structures such as nanowires, nanopods, or outer membrane tubes (Shetty et al. 2011, Wei et al. 2014, Barchinger et al. 2016). Bacterial nanotubes emanate directly from the cell membrane and comprise a lipid bilayer having a continuous lumen (Dubey et al. 2016). These membrane-derived extensions have been suggested to mediate intercellular material exchange, although their physiological significance remains debated (Pospíšil et al. 2020). While the relevance of such structures to sporulation has not been demonstrated, they highlight the possibility that close physical continuity between neighboring cells can facilitate communication.

In this study, we reconstituted the intercellular signal transduction of SpoIIR by co-culturing a *spoIIR*^+^ Δ*spoIIGA* strain (Donor of SpoIIR signal) with a Δ*spoIIR spoIIGA*^+^ (Acceptor of SpoIIR signal). Using this system, we showed that extracellular materials, including secreted proteins and membrane vesicles, did not appear to be responsible for SpoIIR signaling. Cessation of co-incubation shaking improved SpoIIR signal transduction, while addition of membrane-solubilizing detergent abolished it. Secretion or extracellular translocation of SpoIIR was not required for signaling under normal sporulation conditions, since N-terminal hydrophobic domain of SpoIIR was interchangeable with N-terminal membrane spanning domain whose C-terminus is known to be in cytoplasm. In addition, the N-terminal signal peptide-like domain of SpoIIR was dispensable upon SpoIIR expression only in the mother cell. Overall, these results indicate that SpoIIR is not extracellularly secreted but transferred from the forespore into the mother cell cytoplasm and interacts with the cytoplasmic domain of SpoIIGA to trigger pro-σ^E^ processing (see Fig. 1b).

## Results

### Signal transduction between co-cultured *B. subtilis*-sporulating cells

**Figure 2:**
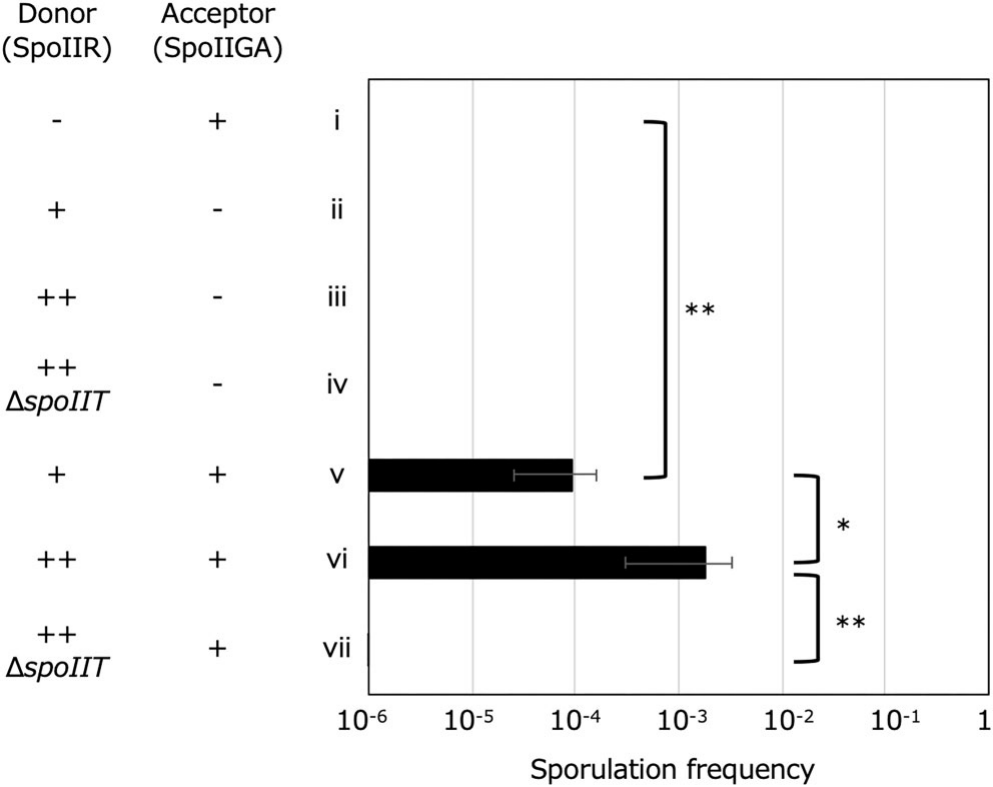
SpoIIR signaling between co-cultured *B. subtilis*-sporulating cells. *Bacillus subtilis* strains of *comK*-deleted, *spoIIR*-deleted and *spoIIGA*-positive strain (NK01) (i), *spoIIR*-positive and *spoIIGA*-deleted strain (NK03) (ii), multicopy *spoIIR*-positive and *spoIIGA*-deleted strain (NK04) (iii), multicopy *spoIIR*-positive, *spoIIGA*-deleted and *spoIIT*-deleted strain (NK05) (iv), NK03/NK01 (v), NK04/NK01 (vi), and NK05/NK01 (vii) were cultured or co-cultured as detailed in the Materials and Methods section and sporulation frequencies at 24 h after the initiation of co-incubation (T_24_) are shown. +/– indicate presence/absence of *spoIIR* or *spoIIGA*, respectively. ++ indicates presence of multicopy *spoIIR*. Δ*spoIIT* indicates deletion of the *spoIIT* gene. The data are the mean of at least three independent experiments. Error bars indicate standard deviations. Significance was calculated using t test comparing indicated samples. * *P* value < 0.05, ** *P* value < 0.01. Table S2 contains the CFU of viable cells, spores, and sporulation frequencies.

We first attempted to reconstitute SpoIIR signal transduction between co-cultured cells based on the approach by Diez and colleagues (Diez et al. 2012). In their study, two mutant strains of *B. subtilis* were co-cultured: a *spoIIR*-positive but *spoIIGA*-deleted strain (serving as the donor of the SpoIIR signal) and a *spoIIR*-deleted but *spoIIGA*-positive strain (serving as the acceptor). Because SpoIIR is produced only in the donor strain and SpoIIGA only in the acceptor, activation of SpoIIGA-dependent pro-σ^E^ processing observed in the acceptor strain should be the result of intercellular signaling of SpoIIR. Diez et al. observed spore formation under these mixed-culture conditions, indicating that SpoIIR produced in donor cells was capable of transducing the signal to SpoIIGA in acceptor cells, suggesting the intercellular signaling via SpoIIR. To further confirm this conclusion, we introduced *comK* deletion to eliminate the possibility that the *spoIIR* gene was transferred to the acceptor cells by natural transformation. As shown in Fig. 2, the single cultures of donor and acceptor strains were Spo^−^ (Fig. 2i and ii), while the mixed culture produced heat-resistant spores (Fig. 2v). The observed erythromycin resistance and chloramphenicol susceptibility in all spores confirmed the sporulation of the acceptor cells, not the donor cells, following co-incubation with the SpoIIR signal-bearing donor cells. The colonies arising from heat-resistant spores were Spo^−　^suggesting that sporulation in acceptor cells induced by co-incubation with the donor strain occurred independently of genetic alterations, including transformation and suppressor mutations. Under phase contrast microscopy, the spores formed in the co-incubation culture exhibited normal morphology compared with those from the wild-type culture (Fig. S1). A *spoIIR* gene with its native promoter was introduced into a multicopy vector to increase SpoIIR expression in the donor strain. The strain with overexpressed SpoIIR showed heightened sporulation in the acceptor strain, suggesting the *spoIIR* expression dependence of signaling from the donor to acceptor strain (Fig. 2iii and vi). Since SpoIIR accumulates at too low a level to detect (Londoño-Vallejo and Stragier 1995), it is not confirmed if SpoIIR is overexpressed by increased copy number and the SpoIIR expression is responsible for the signaling. To confirm whether the SpoIIR expression is responsible for the signaling, *de novo* protein synthesis was inhibited in the donor cells by adding the translation inhibitor erythromycin at several time points during sporulation. This treatment does not affect erythromycin-resistant acceptor cells (*B. subtilis* NK01; Δ*spoIIR::erm* Δ*comK::kan*). Signal transduction was abolished only when *de novo* protein synthesis was inhibited at T_2_ (2 h after the initiation of sporulation), the time of *spoIIR* expression, or earlier (Fig. S2). These results indicate that signal transmission from the donor to the acceptor cells occurred through protein expression in the donor cells at T_2_, which includes SpoIIR. We next confirmed the effect of *spoIIT* deletion on the SpoIIR signaling. SpoIIT is expressed in the forespore and is a co-activator of SpoIIGA-dependent pro-σ^E^ processing (Meeske et al. 2016). The abolition of SpoIIGA activation (Fig. 2vii) upon deletion of the *spoIIT* gene in the donor strain suggests that the signal transduction facilitated by SpoIIR between the co-cultured donor and acceptor strains operates via a mechanism similar to that between an adjacent forespore and mother cell during endosporulation.

### Neither secreted proteins nor membrane vesicles are responsible for SpoIIR signaling

**Figure 3:**
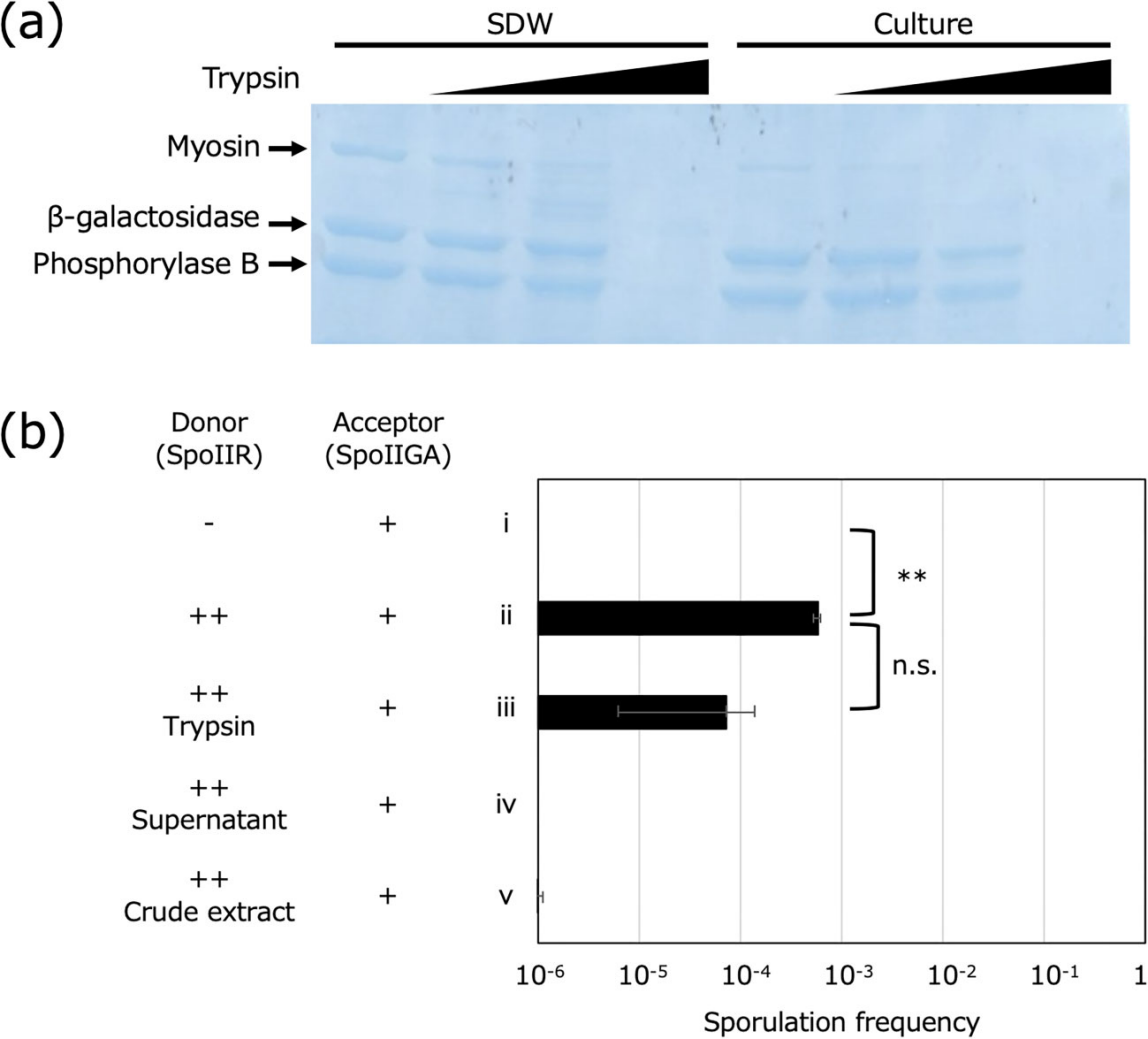
SpoIIR signaling is not mediated by extracellular proteins or whole-cell extracts. a; Protein molecular weight markers (Takara Bio Inc.), including pig myosin (200 kDa), *E. coli* β-galactosidase (116 kDa), and rabbit muscle phosphorylase B (97.2 kDa), were added to sterile distilled water (SDW) or *B. subtilis* culture supernatant at T_2_ (Culture) to the concentration of 0.9 µg/µl. Serially increased trypsin values (0, 0.025, 0.05, or 0.1%), as indicated by triangles, were added and incubated at 37°C for 2 h. Proteins were separated by SDS-polyacrylamide gel electrophoresis and stained with Coomassie brilliant blue. b; *B. subtilis* strains of *comK*-deleted, *spoIIR*-deleted and *spoIIGA*-positive strain (NK01) and multicopy *spoIIR*-positive and *spoIIGA*-deleted strain (NK04) were co-cultured as detailed in the Materials and Methods section and sporulation frequencies at 24 h after the initiation of co-incubation (T_24_) are shown (ii). −, + and ++ indicate absence of *spoIIR*, presence of *spoIIGA* and presence of multicopy *spoIIR*, respectively. 0.1% trypsin was added at the initiation of sporulation (iii). *B. subtilis* NK01 and NK04 were induced to sporulate for 2.5 h (T_2.5_) in a sporulation medium. The NK01 cell pellet from 5 ml culture was resuspended with 5 ml sporulation medium (i) or culture supernatant of NK04 passed through a 0.2 um filter (iv) and continued incubation until T_24_. French-pressured crude cell extracts of *B. subtilis* NK04 at T_2.5_ (v) was added into NK01 culture at T_2.5_, as indicated in the Materials and Methods section. The data are the mean of at least three independent experiments. Error bars indicate standard deviations. Significance was calculated using t test comparing indicated samples. ** *P* value < 0.01, n.s. not significant. Table S2 contains the CFU of viable cells, spores, and sporulation frequencies.

Whereas SpoIIR possesses an N-terminal signal peptide-like hydrophobic domain (ND_SpoIIR_), it was detected in the cell envelope fraction (Imamura et al. 2008) and its cleavage product, indicative of secretion, was not observed (Imamura et al. 2008, Diez et al. 2012, Londoño-Vallejo 1997a). In addition, as SpoIIR can transduce the signal from the forespore to the mother cell without its release from the membrane (Londoño-Vallejo 1997a), we investigated whether SpoIIR is secreted under our co-culture conditions. Because SpoIIR protein was not detectable due to the extremely low accumulation (Londoño-Vallejo and Stragier 1995), externally supplied proteins, including pig myosin, *E. coli* β-galactosidase, and rabbit muscle phosphorylase B, were used to confirm the protease activity in the culture. These proteins were stable in water but degraded upon addition of protease trypsin (Fig. 3a). These proteins were slightly degraded in the *B. subtilis* culture supernatant at T_2_, probably due to the extracellular protease secreted from *B. subtilis* cells. The retained proteins in the culture supernatant were degraded upon trypsin addition, indicating that 0.1% trypsin was functional and sufficient to degrade proteins in the sporulating *B. subtilis* culture (Fig. 3a). Therefore, 0.1% trypsin was added to the co-incubating culture of the donor and acceptor strains to determine whether secreted proteins are involved in the signaling. However, trypsin addition did not abolish SpoIIR signaling between co-cultured cells (Fig. 3b ii and iii). These results suggest that intercellular signaling is resistant to degradation by extracellular proteases.

We hypothesized that SpoIIR might be transferred from the forespore to the mother cell via membrane vesicles without being released from the membrane. To test this hypothesis, acceptor cells were resuspended with a filtered culture supernatant of *B. subtilis* donor cells at T_2.5_. However, the culture supernatants failed to induce sporulation in the acceptor strain (Fig. 3iv). To efficiently produce membrane vesicles, *B. subtilis* cells expressing SpoIIR was passed twice through a French pressure cell, sonicated to produce a mixture of inside-out and right-side-out vesicles, and the crude lysate was added to the acceptor cell culture. However, the crude cell extracts did not transduce the signal, as in the case of co-cultivation (Fig. 3v). While these results suggest that donor cell-produced membrane vesicles do not transfer SpoIIR into the acceptor cells, they also indicate that dead cells or cell debris were unable to transduce the SpoIIR signal. Overall, we observed SpoIIR signaling between co-cultured donor and acceptor cells but the signaling mechanism does not appear to involve secretion, membrane vesicles or cell debris.

### Possible signaling passway of SpoIIR

**Figure 4:**
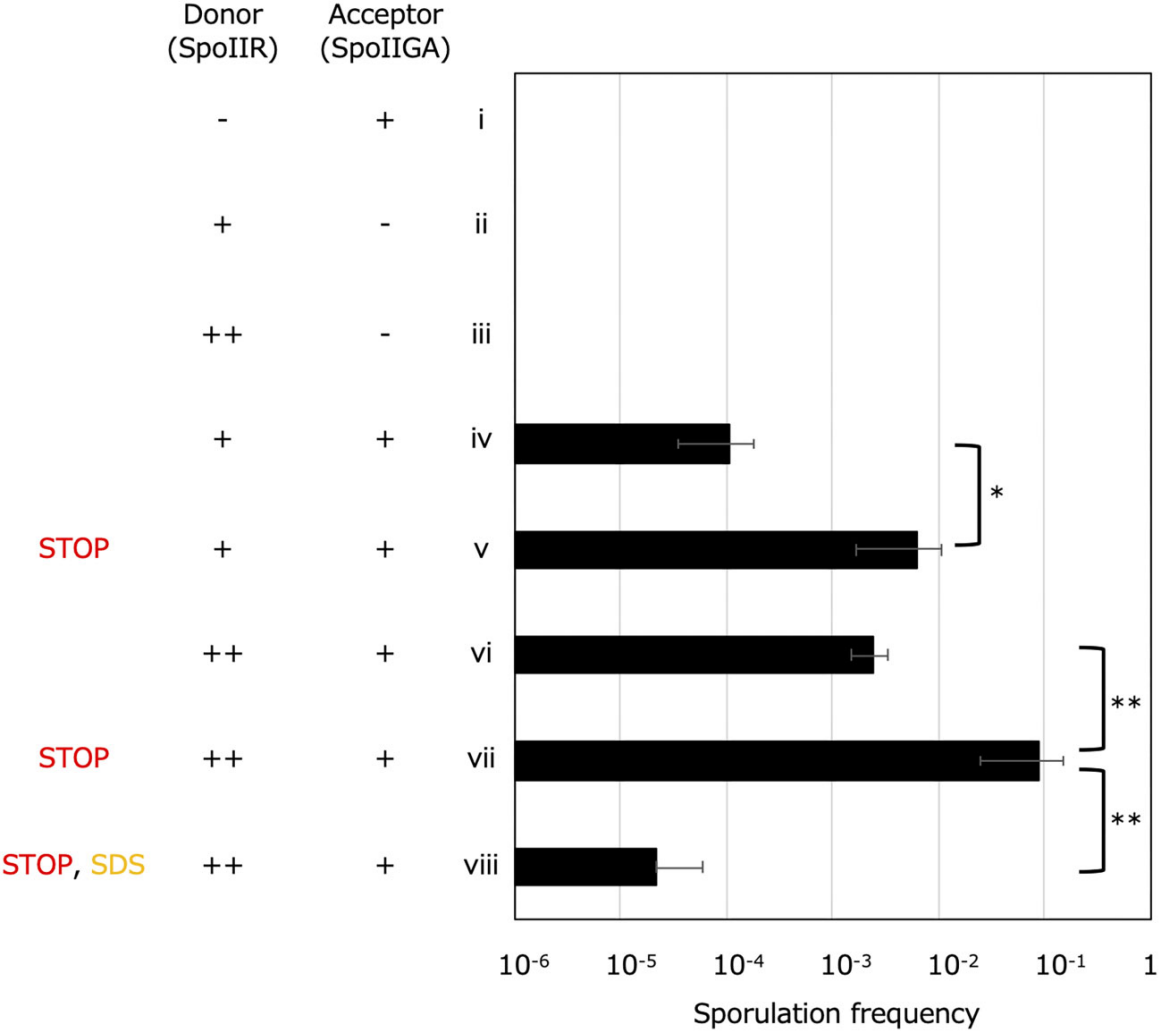
Effects of ceasing shaking and SDS on SpoIIR signaling between co-cultured cells. Individual incubation or co-incubation of *comK*-deleted, *spoIIR*-deleted and *spoIIGA*-positive *B. subtilis* strain (NK01) (i), *spoIIR*-positive and *spoIIGA*-deleted *B. subtilis* strain (NK03) (ii), multicopy *spoIIR*-positive and *spoIIGA*-deleted *B. subtilis* strain (NK04) (iii), NK03/NK01 (iv, v), and NK04/NK01 (vi–viii) with NK01 (ix–xi) were performed as detailed in the Materials and Methods section and sporulation frequencies at 24 h after the initiation of co-incubation (T_24_) are shown. +/– indicate presence/absence of *spoIIR* or *spoIIGA*, respectively. ++ indicates presence of multicopy *spoIIR*. Δ*spoIIT* indicates deletion of the *spoIIT* gene. Culture shaking was stopped at T_2_ for 1 h (v, vii, viii). 0.006% SDS was added at T_0_ (viii). The data are the mean of at least three independent experiments. Error bars indicate standard deviations. Significance was calculated using t test comparing indicated samples. * *P* value < 0.05, ** *P* value < 0.01. Table S2 contains the CFU of viable cells, spores, and sporulation frequencies.

The data presented above suggest the non-involvement of secreted proteins and membrane vesicles in SpoIIR signaling. Thus, we next hypothesized that SpoIIR is transferred from the forespore into the mother cell without secretion (Fig. 1b). One of the possibilities of direct transfer from the forespore into the mother cell is membranous nanotubes. It is conceivable that SpoIIR moves from the forespore to the mother cell through the continuous fluidic membrane without secretion. If this is the case, membrane structure between co-cultured cells should be stabilized by ceasing shaking during incubation, thereby increasing the efficiency of SpoIIR signal transduction. In co-cultivation with both donor strains—*spoIIR*-expressing and *spoIIR*-overexpressing *B. subtilis*—sporulation frequencies of the acceptor strain increased approximately 50-fold upon cessation of culture shaking at T_2_ for 1 h, which is consistent with the hypothesis (Fig. 4iv–vii). The sensitivity of the signal transduction to the membrane-solubilizing detergent, sodium dodecyl sulfate (SDS), was tested as used in a previous study to investigate the nature of nanotubes (Dubey and Ben-Yehuda 2011). At a concentration of 0.006% SDS, sporulation frequency drastically decreased in co-culture, yet the viabilities of all donor and acceptor cells remained unaltered (Fig. 4viii). *Bacillus subtilis*-wild type strain168 has an integrative and conjugative element (ICE*Bs1*), which drives horizontal gene transfer by conjugation (Suzuki et al. 2020). ICE*Bs1* was deleted from both donor and acceptor strains but the donor strain successfully activated SpoIIGA in the acceptor cells (Fig. S3), confirming that conjugation is not involved in SpoIIR signaling. These results are consistent with the idea that SpoIIR is transferred into the mother cell compartment through membranes.

### SpoIIR lacking its signal peptide-like domain allows pro-σ^E^ activation when produced only in the mother cell compartment

**Figure 5:**
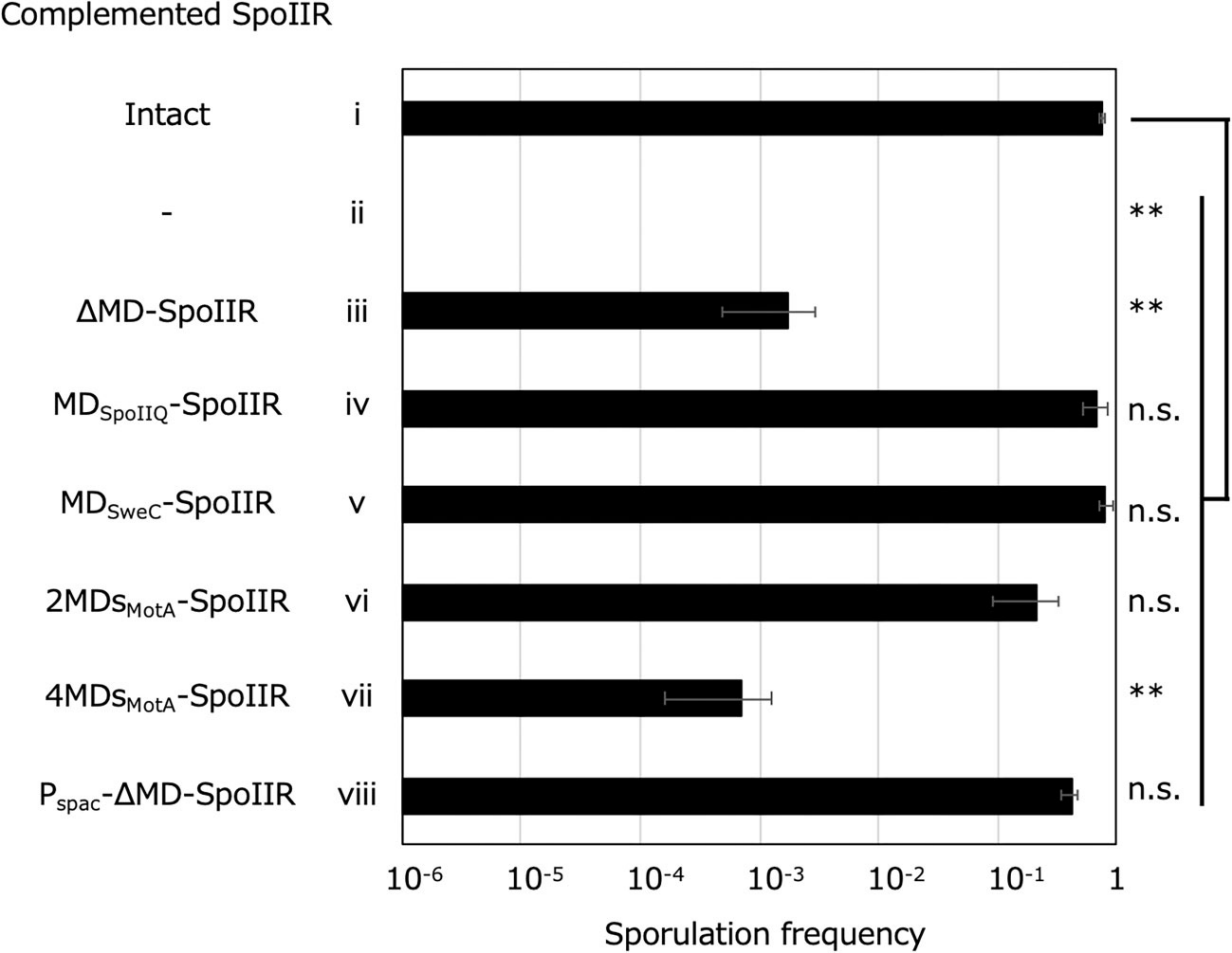
Requirements of the N-terminal domain of SpoIIR for signaling. SpoIIR proteins indicated at left were expressed from the *thrC* locus during *B. subtilis* sporulation. Genes encoding the following proteins were introduced into the *thrC* locus of the *spoIIR* deletion strain under control of the native *spoIIR* promoter unless indicated otherwise, intact SpoIIR (NK06) (i), none (BKE36970) (ii), SpoIIR without the N-terminal domain (NK07) (iii), SpoIIR, whose N-terminal domain was replaced by the MD of SpoIIQ (NK08) (iv), the MD of SweC (NK09) (v), 2 MDs (the first and the second) of MotA (NK10) (vi), or 4 MDs of MotA (NK11) (vii), and SpoIIR without the N-terminal domain under control of the spac promoter (NK12) (viii). These strains were induced to sporulate in DS medium. Sporulation frequencies at T_24_ are shown. The data are the mean of at least three independent experiments. Error bars indicate standard deviations. Significance was calculated using dunnett’s test comparing with intact *spoIIR* (i) and other samples (ii-viii). ** *P* value < 0.01, n.s. not significant. Table S2 contains the CFU of viable cells, spores, and sporulation frequencies.

The above co-culturing experiments suggest that SpoIIR transduces a signal without secretion. However, the co-culturing signal transduction represents an artificial condition, leaving open the possibility that SpoIIR expressed in donor cells may transferred to the acceptor cell’s forespore (rather than its mother cell), ultimately transmitting the signal to the mother cell via an unknown mechanism. In order to investigate the necessity of SpoIIR secretion during natural sporulation, the importance of signal peptide-like domain of SpoIIR was tested. It was shown previously that a chimeric version of SpoIIR, in which the N-terminal signal peptide-like domain was replaced by the N-terminal membrane domain of SpoIIQ (MD_SpoIIQ_-SpoIIR), fully complemented sporulation of the Δ*spoIIR* strain, indicating the dispensability of SpoIIR release from the membrane in signal transduction (Londoño-Vallejo 1997a). SpoIIQ is translocated to the surface of the inner membrane when produced in *E. coli* cells and presumably to the surface of the forespore membrane during *B. subtilis* sporulation (Londoño-Vallejo et al. 1997b). Thus, we investigated the necessity of extracellular translocation of SpoIIR to the surface of the forespore membrane by introducing several versions of *spoIIR* into the *thrC* locus of the Δ*spoIIR* strain. Full-length *spoIIR* with its native promoter fully complemented the sporulation deficiency of the Δ*spoIIR* strain (Fig. 5i and ii). Deleting the N-terminal signal peptide-like domain of SpoIIR did not eliminate sporulation but markedly impaired it (Fig. 5iii). This result suggests that SpoIIR signaling can occur at low frequency during sporulation without the SpoIIR N-terminal hydrophobic domain, maybe by diffusion from the forespore to the mother cell through the lumen of nanotubes. The MD of SpoIIQ was fully functional, consistent with a previous report (Fig. 5iv) (Londoño-Vallejo 1997a). SweC and MotA are well-characterized membrane proteins with 1 and 4 N-terminal membrane-spanning domains, respectively, whose C-termini are located in the cytoplasm (Brunet et al. 2019, Santiveri et al. 2020). The chimeric protein MD_SweC_-SpoIIR was fully functional regarding induction of sporulation (Fig. 5v). The 2 (i.e. lacking TMD 3 and 4) or 4 transmembrane-spanning domain chimeras of MotA fused to SpoIIR were also tested. The 2 MD chimera almost fully complemented (Fig. 5vi), but the 4 MD chimera impaired SpoIIR function (Fig. 5vii) to a level comparable to that of SpoIIR without the N-terminal domain (Fig. 5iii), suggesting that an increased MD number may hinder SpoIIR fluidity within membrane. It is not clear why MD of both SpoIIQ and SweC, in which the C-terminal SpoIIR domain was expected to be translocated outside or remain within the forespore, respectively, allowed the signal transduction. Perhaps the MD of SpoIIQ fails to translocate all SpoIIR molecules. Although the actual insertions and translocation of chimeric proteins in the forespore membrane were not experimentally verified, the results with SpoIIR lacking its signal peptide-like domain, with MD_SweC_-SpoIIR, and with 2MD_MotA_-SpoIIR support the idea that SpoIIR can trigger SpoIIGA-dependent pro-σ^E^ processing during sporulation without being released from the forespore membrane and independent of extracellular translocation.

**Figure 6:**
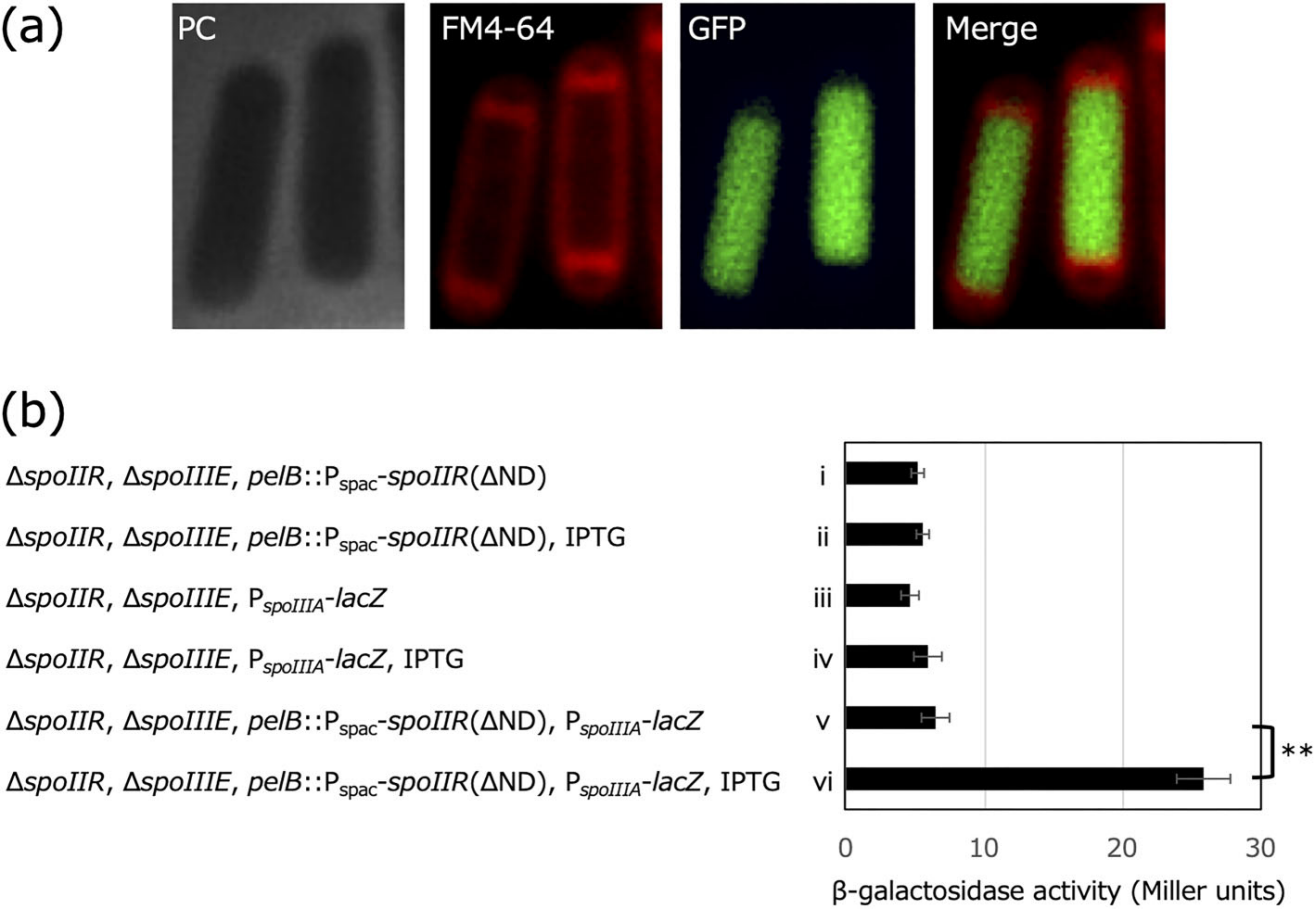
Signal transduction of SpoIIR specifically expressed in the mother cell. a; Mother cell-specific expression of GFP from the *pelB* locus in the *B. subtilis* Δ*spoIIR* and Δ*spoIIIE* mutant strain NK18. Expression of GFP from the spac promoter (P_spac_) was induced by IPTG addition. Phase contrast image (PC), membrane-stained fluorescence image (FM4-64), GFP image (GFP), and merged FM4-64 and GFP fluorescence image (Merge) are shown. b; Activation of σ^E^ by SpoIIR lacking its N-terminal domain, produced in the mother cell compartment. Strains carrying Δ*spoIIR* and Δ*spoIIIE* mutations NK19 (i and ii), NK22 (iii and iv) and NK20 (v and vi) were induced to sporulate. Some strains contained the IPTG-inducible gene encoding SpoIIR lacking its N-terminal domain at the *pelB* locus, designated *pelB*::P_spac_-*spoIIR*(ΔND) (i, ii, v, and vi). σ^E^ activity was measured using the σ^E^-dependent *spoIIIA* promoter fused to the *lacZ* reporter gene, designated P*_spoIIIA_*-*lacZ* (iii-vi). 1 mM IPTG was added at T_2.5_ (ii, iv and vi), cells were harvested at T_4.5_ and subjected to the β-galactosidase assay. Average Miller units of three independent experiments were shown. Error bars indicate standard deviations. Significance was calculated using t test comparing indicated samples. ** *P* value < 0.01.

SpoIIR’s interaction with SpoIIGA expressed in *E. coli* has been established (Imamura et al. 2008), and the extracellular translocation of SpoIIR appears to be dispensable in signal transduction (Fig. 5). Therefore, SpoIIR may move from the forespore to the mother cell compartment to interact with the cytoplasmic domain of SpoIIGA (Fig. 1b), instead of with its extracellular domains as previously proposed (Fig. 1a). If this is the case, SpoIIR lacking the N-terminal hydrophobic domain should be able to induce pro-σ^E^ processing when produced in the mother cell cytoplasm. SpoIIR lacking the N-terminal domain expressed from the spac promoter, which induces uncompartmentalized transcription, complemented the sporulation defects of the Δ*spoIIR* strain (Fig. 5viii), consistent with a previous report (Londono-Vallejo 1997a). These results may indicate that SpoIIR can trigger SpoIIGA-dependent pro-σ^E^ processing within the mother cell cytoplasm. However, these experiments expressed SpoIIR in both compartment and thus, it is not clear whether the increased sporulation frequency is due to the SpoIIR produced in the mother cell or forespore. To establish mother cell-specific expression of *spoIIR*, the gene encoding DNA translocase SpoIIIE was deleted in the Δ*spoIIR* strain. As expected, the Δ*spoIIIE*, Δ*spoIIR* strain exhibited a disporic phenotype, forming two forespores, one at each cell pole. The two copies of the chromosome produced upon starvation of this mutant are stuck with the origin proximal one-third in the forespore and origin distal two-thirds, including replication terminus, in the mother cell (Burton et al. 2007). IPTG-inducible *gfp* or *spoIIR* without the region encoding the N-terminal hydrophobic domain, P_spac_-*gfp* or P_spac_-*spoIIR*(ΔND), respectively, was integrated at the *pelB* locus near the replication terminus region that localizes absolutely in the mother cell of the *spoIIIE* mutant. Hence, expression of the genes integrated at the *pelB* locus was inducible specifically in the mother cell. As expected, GFP fluorescence from the P_spac_-*gfp* containing strain was observed only in the mother cell compartment, indicating that the system successfully achieved mother cell-specific gene expression (Fig. 6a). We introduced a reporter of σ^E^ activity, P*_spoIIIA_*-*lacZ*, into the P_spac_-*spoIIR*(ΔND), Δ*spoIIIE* strain to test whether SpoIIR(ΔND) produced only in the mother cell is capable of triggering pro-σ^E^ processing by SpoIIGA. As shown in Fig. 6b, SpoIIR(ΔND) expressed only in the mother cell compartment under the Δ*spoIIIE* background allowed σ^E^ activation, indicating that SpoIIR lacking its N-terminal signal peptide-like hydrophobic domain signals to SpoIIGA within the mother cell compartment.

## Materials and methods

### Construction of bacterial strains and plasmids

Bacterial strains and plasmids used in this study are listed in Table 1. The oligonucleotide primers used in this study are listed in Table S1. All *B. subtilis* strains used in this study were derivatives of the wild-type strain 168. Strains were constructed by transformation using plasmids or genomic DNA as indicated in Table 1.

**Table 1:** Bacterial strains and plasmids used in this study.

Strains	Genotype/description	Source/reference^a^
*Bacillus subtilis*		
168	*trpC2*	Laboratory stock
BKE36970	*trpC2* Δ*spoIIR::erm*	Koo et al. 2017
BKE36770	*trpC2* Δ*spoIIT::erm*	Koo et al. 2017
BKE16800	*trpC2* Δ*spoIIIE::erm*	Koo et al. 2017
8G32	*trpC2* Δ*comK::kan*	Ogura and Tanaka 1997
NK01	*trpC2* Δ*spoIIR::erm* Δ*comK::kan*	8G32→BKE36970
NK02	*trpC2* Δ*spoIIGAB::cat*	This work
NK03	*trpC2* Δ*spoIIGAB::cat aprE*::P*_spoIIG_-spoIIGB spec*	pNK01→NK02
NK04	*trpC2* Δ*spoIIGAB::cat aprE*::P*_spoIIG_-spoIIGB spec* pHY300PLK::P*_spoIIR_*-*spoIIR tet*	pNK02→NK03
NK05	*trpC2* Δ*spoIIGAB::cat aprE*::P*_spoIIG_-spoIIGB spec* pHY300PLK::P*_spoIIR_*-*spoIIR tet* Δ*spoIIT::erm*	BKE36770→NK04
NK06	*trpC2* Δ*spoIIR::erm thrC*::P*_spoIIR_*-*spoIIR cat*	pNK06→BKE36970
NK07	*trpC2* Δ*spoIIR::erm thrC*::P*_spoIIR_*-*spoIIR*(24–224) *cat*	pNK07→BKE36970
NK08	*trpC2* Δ*spoIIR::erm thrC*::P*_spoIIR_*-MD*_spoIIQ_-spoIIR*(24–224) *cat*	pNK08→BKE36970
NK09	*trpC2* Δ*spoIIR::erm thrC*::P*_spoIIR_*-MD*_sweC_-spoIIR*(24–224) *cat*	pNK09→BKE36970
NK10	*trpC2* Δ*spoIIR::erm thrC*::P*_spoIIR_*-MD*_motA_*(1–58)-*spoIIR*(24–224) *cat*	pNK10→BKE36970
NK11	*trpC2* Δ*spoIIR::erm thrC*::P*_spoIIR_*-MD*_motA_*(1–208)-*spoIIR*(24–224) *cat*	pNK11→BKE36970
NK12	*trpC2* Δ*spoIIR::erm thrC*::P_spac_-*spoIIR*(24–224) *cat*	pNK12→BKE36970
NK13	*trpC2* Δ*spoIIR*	This work
NK14	*trpC2* Δ*spoIIR* Δ*spoIIIE::erm*	BKE16800→NK13
NK18	*trpC2* Δ*spoIIR* Δ*spoIIIE::erm pelB*::P_spac_*-gfp*	pNK15→NK14
NK19	*trpC2* Δ*spoIIR* Δ*spoIIIE::erm pelB*::P_spac_*-spoIIR*(24–224)	pNK16→NK14
NK20	*trpC2* Δ*spoIIR* Δ*spoIIIE::erm pelB*::P_spac_*-spoIIR*(24–224) P*_spoIIIA_-lacZ*	pNK20→NK19
NK21	*trpC2* Δ*spoIIR* Δ*spoIIIE::erm pelB*::P_spac_-	pNK14→NK14
NK22	*trpC2* Δ*spoIIR* Δ*spoIIIE::erm pelB*::P_spac_- P*_spoIIIA_*-*lacZ*	pNK20→NK21
ΔICEBs1	*trpC2* Δ*ICEBs1*	This work
NK23	*trpC2* Δ*ICEBs1* Δ*spoIIR::erm*	BKE36970→ΔICEBs1
NK24	*trpC2* Δ*ICEBs1* Δ*spoIIR::erm ΔcomK::kan*	8G32→NK23
NK25	*trpC2* Δ*ICEBs1* Δ*spoIIGAB::cat*	NK02→ΔICEBs1
NK26	*trpC2* Δ*ICEBs1* Δ*spoIIGAB::cat aprE*::P*spoIIG-spoIIGB spec*	pNK01→NK25
NK27	*trpC2* Δ*ICEBs1* Δ*spoIIGAB::cat aprE*::P*spoIIG-spoIIGB spec* pHY300PLK::P*spoIIR-spoIIR tet*	pNK02→NK26
*E. coli*		
DH5α	F^−^ Φ80d*lacZ*ΔM15 Δ(*lacZYA-argF*)U169 *deoR recA*1 *endA*1 *hsdR*17(rk^−^ mk^+^) *phoA supE*44 λ^−^*thi*-1 *gyrA*96 *relA*1	Laboratory stock
Plasmids		
pMF20	*amyE*-N *gfp cat amyE*-C *bla*	Murakami et al. 2002
pAPNC213	*aprE*-N *aprE*-C *spec*	Morimoto et al. 2002
pHY300PLK	*tet bla*	Takara
pET21a	*bla lacI*	Invitrogen
pTCC1	*thrCN cat thrCC bla*	Imamura et al. 2004
pMUTinT3	*lacZ lacI bla erm*	Kobayashi et al. 2003
pDGREF	*bla spc xylR* P*_xyl_-mazF*	Yu et al. 2010
pNK01	*aprE*-N *aprE*-C *spec* P*_spoIIG_-spoIIGB*	This work
pNK02	*tet bla* P*_spoIIR_-spoIIR*	This work
pNK06	*thrCN cat thrCC bla* P*_spoIIR_*-*spoIIR*	This work
pNK07	*thrCN cat thrCC bla* P*_spoIIR_-spoIIR*(24–224)	This work
pNK08	*thrCN cat thrCC bla* P*_spoIIR_*-MD*_spoIIQ_-spoIIR*(24–224)	This work
pNK09	*thrCN cat thrCC bla* P*_spoIIR_*-MD*_sweC_-spoIIR*(24–224)	This work
pNK10	*thrCN cat thrCC bla* P*_spoIIR_*-MD*_motA_*(1–58)-*spoIIR*(24–224)	This work
pNK11	*thrCN cat thrCC bla* P*_spoIIR_*-MD*_motA_*(1–208)-*spoIIR*(24–224)	This work
pNK12	*thrCN cat thrCC bla* P_spac_-*spoIIR*(24–224)	This work
pNK13	*pelB*-N *aprE*-C *spec*	This work
pNK14	*pelB*-N *pelB*-C *spec*	This work
pNK15	*pelB*-N *pelB*-C *spec* P_spac_-*gfp*	This work
pNK16	*pelB*-N *pelB*-C *spec* P_spac_-*spoIIR*(24–224)	This work
pNK17	*bla erm lacI lacZ* P_spac_*-mazF*	This work
pNK18	*lacZ lacI bla erm mazF spoIIR*(UP) *spoIIR*(DOWN)	This work
pNK19	*lacZ lacI bla cat*	This work
pNK20	P*_spoIIIA_-lacZ lacI bla cat*	This work

a Arrows indicate direction of transformation from donor DNA to the recipient strain.

To construct deletion strain of *spoIIGA spoIIGAB*(*sigE*) operon (NK02), the primer pairs of P1/P2, P3/P4, and P5/P6, were used to PCR amplify upstream and downstream regions of *spoIIG* operon from *B. subtilis* genomic DNA, and chloramphenicol resistant cassette *cat* from pMF20, respectively. The amplified fragments were ligated in order of upstream-*cat*-downstream of *spoIIG* operon by overlap extension PCR　(Hilgarth and Lanigan 2019). The resulting DNA fragment was used for transformation of *B. subtilis* 168 to replace *spoIIG* operon with *cat* by double crossover generating NK02.

Plasmids were constructed in *E. coli* DH5α, and their structures were confirmed by PCR and DNA sequencing.

Whole pAPNC213 was amplified by inverse PCR using primer pair of P7/P8. Promoter region of *spoIIG* operon and *sigE* gene fragment were PCR amplified using primer pairs of P9/P10 and P11/P12, respectively and ligated by overlap extension PCR. The P*_spoIIG_*-*sigE* fragment was cloned into the pAPNC213 fragment by Gibson assembly (New England BioLabs) to generate pNK01.

P*_spoIIR_*-*spoIIR* fragment was PCR amplified using primer pair of P13/P14. pHY300PLK was digested by *Bgl*II/*Bam*HI and treated with alkaline phosphatase. *Bgl*II/*Bam*HI digested P*_spoIIR_*-*spoIIR* fragment was cloned into the pHY300PLK to generate pNK02.

P*_spoIIR_*-*spoIIR* fragment was PCR amplified using primer pair of P23/P14, digested with *Pst*I/*Bam*HI, and cloned into *Pst*I/*Bgl*II-digested pTCC1 to generate pNK06. P*_spoIIR_* and *spoIIR* without the region coding N-terminal domain were amplified using primer pairs of P23/P25 and P24/P14, respectively, and ligated by overlap extension PCR. The fragment was digested with *Pst*I/*Bam*HI and cloned into *Pst*I/*Bgl*II-digested pTCC1 to generate pNK07. P*_spoIIR_*, the region encoding membrane domain of SpoIIQ, *spoIIR* without the region coding N-terminal domain were amplified using primer pairs of P25/P23, P26/P27, and P28/P14, respectively, and ligated by overlap extension PCR. The fragment was digested with *Pst*I/*Bam*HI and cloned into *Pst*I/*Bam*HI-digested pTCC1 to generate pNK08. pNK06 without the region encoding N-terminal domain of SpoIIR was amplified by inverse PCR using primer pair of P25/P28. The region encoding membrane domain of SweC was amplified using primer pairs of P29/P30 and ligated with the pNK06 fragment by Gibson assembly to generate pNK09.

P*_spoIIR_*, the region encoding 4 membrane spanning domains of MotA, *spoIIR* without the region coding N-terminal domain were amplified using primer pairs of P25/P23, P32/P33, and P28/P14, respectively and ligated by overlap extension PCR. The fragment was digested with *Pst*I/*Bam*HI and cloned into *Pst*I/*Bgl*II-digested pTCC1 to generate pNK11. pNK11 without the region encoding second and third membrane spanning domains of MotA was amplified by inverse PCR using primer pair of P31/P28 and self-ligated to generate pNK10.

P_spac_ promoter of pMUTinT3, SD region of *spoIIR*, and *spoIIR* without the region coding N-terminal domain were amplified using primer pairs of P34/P35, P36/P25, and P28/P37, respectively and ligated by overlap extension PCR. *lacI* of pMUTinT3 was amplified using primer pair of P38/P39. *Pst*I/*Xba*I-digested P_spac_-SD-*spoIIR* (without N-terminal domain) fragment, *Xba*I/*Bam*HI-digested *lacI* fragment, and *Pst*I/*Bgl*II-digested pTCC1 were ligated to generate pNK12.

Whole pAPNC213 without *aprE*-N was amplified by inverse PCR using primer pair P40/P41. The region encoding N-terminal PelB was PCR amplified using primer pair P42/P43 and cloned into the pAPNC213 fragment by Gibson assembly to generate pNK13. Whole pNK13 without *aprE*-C was amplified by inverse PCR using primer pair P44/P45. The region encoding C-terminal PelB was PCR amplified using primer pair P46/P47 and cloned into the pNK13 fragment by Gibson assembly to generate pNK14. Whole pNK14 was amplified by inverse PCR using primer pair P48/P49. Fragments of *gfp* and *spoIIR* without the region coding N-terminal domain were PCR amplified from pMF20 and *B. subtilis* 168 chromosome using primer pairs P50/P51 and P52/P53, then cloned into the downstream of P_spac_ promoter in the pNK14 fragment by Gibson assembly to generate pNK15 and pNK16, respectively.

pMUTinT3 without *lacZ* gene was amplified by inverse PCR using primer pair P54/P55. *E. coli mazF* gene, a counter-selectable marker in *B. subtilis* (Morimoto et al. 2009), was PCR amplified using primer pair P56/P57 from pDGREF and cloned into the downstream of P_spac_ promoter in the pMUTinT3 fragment by Gibson assembly to generate pNK17. Whole pNK17 was amplified by inverse PCR using primer pair P58/P59. Upstream and downstream regions of *spoIIR* were PCR amplified using primer pair of P60/P61 and P62/P63, respectively and cloned into pNK17 fragment by Gibson assembly to generate pNK18. pNK18 was introduced into *B. subtilis* 168 by selecting erythromycin-resistant clones, followed by counter-selection by lethality of *mazF* in *B. subtilis* under the presence of sucrose to generate marker-free *spoIIR* deletion strain NK13.

Whole pMUTinT3 without *erm* gene was amplified by inverse PCR using primer pair P64/P65. Chloramphenicol resistant gene fragment, *cat*, was PCR amplified using the primer pair P66/P67 and pMF20 as a template, followed by ligation using a seamless ligation-independent cell lysate (Zhang et al. 2012) with pMUTinT3 fragment to generate pNK19. Promoter region of *spoIIIA* operon was PCR amplified using primer pair P68/P69. *Sma*I/*Hin*dIII digested *spoIIIA* promoter fragment and *Nru*I/*Hin*dIII digested pNK19 were ligated to generate pNK20.

The ΔICE*Bs1* strain was constructed by amplifying a DNA fragment from the *xis*_ICE_*_Bs1_* to the *ydzL* genes using the primers P70/P71. This fragment was digested with *Hin*dIII and *Bam*HI, then ligated into the *Hin*dIII/*Bam*HI sites of the linearized pMutinT3 plasmid. The resulting plasmid was used to transform *B. subtilis* 168, which was then selected for erythromycin resistance on LB agar plates. This generated a strain in which *xis*_ICE_*_Bs1_* is inducible by the P_spac_ promoter. This strain was spread onto LB agar plates containing 1 mM IPTG, but not erythromycin, and incubated for 16 h to induce the expression of *xis*_ICE_*_Bs1_*, which consequently led to the excision of ICE*Bs1* together with the pMutinT3 plasmid. The resulting single colonies were inoculated onto LB agar plates of both with and without erythromycin and incubated for 16 h. The resulting erythromycin-sensitive strain was confirmed by PCR as ΔICE*Bs1*.

The correct construction of the strains, including all of the *spoIIGA* and *spoIIR* fragments, was verified by sequencing.

### Measurement of spore counts and sporulation frequencies

*Bacillus subtilis* cells were induced to sporulate using the resuspension method (Sterlini and Mandelstam 1969) or DS medium. Co-incubations were started at the time point of resuspension (initiation of sporulation, T_0_) and incubated at 37°C for 24 h (T_24_). The number of viable cells and heat-resistant (80°C for 10 min) spores per milliliter of culture at T_24_ were determined using the colony-forming unit on LB agar plates.

#### Co-incubations of signal donor and acceptor strains

The indicated SpoIIR signal donor and acceptor strains were precultured in 2 ml LB medium for overnight. Sporulation were induced by resuspension method (Sterlini and Mandelstam 1969). One hundred microliters preculture was inoculated into 5 ml growth medium and incubated at 37°C until OD_600_ of 0.4. The cultures from donor and acceptor strains were mixed with 2.5 ml each and centrifuged at 5000 × g for 5 min. The pellet was resuspended with pre-warmed 5 ml sporulation medium and this timepoint was considered as the initiation of sporulation (T_0_). The incubation was continued for 24 h (T_24_) at 37°C.

Trypsin was added at T_0_ at final concentration of 0.1% and incubated for 24 h (T_24_) at 37°C if indicated.

Erythromycin was added at indicated time points from T_0_ to T_6_ at final concentration of 1 µg/ml and incubated for 24 h (T_24_) at 37°C if indicated.

The culture shaking was ceased for 1 hour from T_2_ to T_3_ at 37°C if indicated, restarted the shaking at T_3_ and incubated for 24 h (T_24_) at 37°C if indicated.

#### Sodium dodecyl sulfate polyacrylamide gel electrophoresis (SDS-PAGE)

*Bacillus subtilis* strains of *comK*-deleted, *spoIIR*-deleted and *spoIIGA*-positive strain (NK01) and multicopy *spoIIR*-positive and *spoIIGA*-deleted strain (NK04) were co-cultured and induced to sporulate. Nine and a half microliters of water or culture supernatant at T_2_ was mixed with 0.5 µl of protein molecular weight markers (Takara Bio Inc.), series of concentrations of trypsin was added, and incubated at 37°C for 2 h. After mixing with 2 × sample buffer (0.125 M Tris-HCl pH 6.8, 4% SDS, 10% 2-mercaptoethanol, 10% sucrose, 0.01% bromophenol blue) and boiled for 3 min, proteins were separated with 10% acrylamide gel and stained with coomassie brilliant blue.

### Preparation of whole-cell extracts

*Bacillus subtilis* NK04 was induced to sporulate in 100 ml of sporulation medium using the resuspension method until T_2.5_. The culture was centrifuged, 80 ml supernatants were removed (i.e. cells were five-fold concentrated), 1 mM Phenylmethanesulfonyl fluoride (PMSF) was added, and cells resuspended in 20 ml were passed twice through a French pressure cell (Otake Japan, 5501-M) at 120 MPa. The lysate was sonicated with a sonifier (Branson Ultrasonics Corporation, S-150D) for 3 s 20 times at 22.5 kHz. *Bacillus subtilis* NK01 was induced to sporulate, and the 4 ml culture at T_2.5_ was mixed with 1 ml crude extract of *B. subtilis* NK04. Cells with crude extract were incubated until T_24_.

### Fluorescence microscopy

*Bacillus subtilis* NK18 (Δ*spoIIR, spoIIIE::erm, pelB*::P_spac_-*gfp*) was induced to sporulate in sporulation medium at 37°C. FM4-64 (1 µg/ml) was added at T_0_ and IPTG (1 mM) was added at T_2.5_. Cells were corrected at T_4.5_, mounted on glass slides and observed using an Olympus IX73 microscope with a 100 × objective lens UPLFN100 × 02PH. Images were captured using a DP23M camera (Olympus) and CellSens Standard (Olympus).

#### β-galactosidase assay

*Bacillus subtilis* strains were induced to sporulate in sporulation medium. IPTG (1 mM) was added at T_2.5_ and cells were harvested at T_4.5_. Activities of β-galactosidase were determined as described by Miller (Miller 1972) using o-nitrophenyl-β-D-galactopyranoside (ONPG) as the substrate. Enzyme-specific activity is expressed as nanomoles of substrate (ONPG) hydrolyzed per milligram per minute.

## Discussion

In this study, we have shown that 1) SpoIIR transduces a signal from co-cultured cells expressing SpoIIR to cells lacking SpoIIR, 2) SpoIIT aids SpoIIR signaling, suggesting that the signaling mechanism between co-cultured cells parallels that between adjacent forespore and mother cell compartment in sporulating cells, 3) extracellular materials, including secreted proteins and membrane vesicles, do not appear to be responsible for the signaling, 4) cessation of shaking at T_2_ improves the signaling and addition of membrane-solubilizing detergent abolishes it 5) extracellular translocation of SpoIIR is dispensable for signaling, and 6) SpoIIR functions without the N-terminal hydrophobic domain when expressed only in the mother cell compartment. These results are consistent with the idea that SpoIIR is produced in the forespore and localizes to the forespore membrane, but is neither secreted nor externally translocated. Instead, SpoIIR seems to move from the forespore into the mother cell cytoplasm and interacts with the SpoIIGA cytoplasmic domain to trigger pro-σ^E^ processing (Fig. 1b).

Although the data presented in this study suggest that SpoIIR is capable of activating SpoIIGA within the mother cell compartment, the mechanism by which SpoIIR is transferred from the forespore to the mother cell remains unclear. Cessation of the culture shaking should allow the cells to interact stably, as they do in biofilms. Since the biofilm matrix includes DNA, protein fragments and other components, there are several possible components to mediate SpoIIR signaling. Cerulenin treatment at the onset of sporulation allowed asymmetric septum generation but prevented σ^E^ activation (Schujman et al. 1998). Cerulenin is a fungal toxin that inhibits the type II fatty acid biosynthesis pathway by inactivating *B. subtilis* FabF, an enzyme β-ketoacyl-acyl carrier protein synthase ΙΙ, which catalyzes fatty acid elongation (Garwin et al. 1980). Phospholipids that form the cell membrane are composed of a hydrophilic head group, phosphate and a glycerol molecule, and two hydrophobic fatty acid tails. The fatty acid in the phospholipids is one of the key factors influence cell membrane fluidity. Interestingly, FabF has been implicated in membrane fluidity control (Mansilla et al. 2004). Diez and coworkers reported that FabF activity is required in the forespore to trigger pro-σ^E^ processing (Diez et al. 2012). These results together with our current findings may explain the necessity of FabF activity for pro-σ^E^ processing during sporulation. The *de novo* fatty acid biosynthesis may be necessary for the transfer of SpoIIR from the forespore to the mother cell or to ensure adequate fluidity of the forespore membrane. Electron microscopic or cryo-electron microscopic observation of membrane structures between the forespore and mother cell, and assessment of forespore membrane fluidity could test these possibilities. In addition, high-resolution microscopy of the strains under different conditions, using fluorescent reporters and tags for both SpoIIR and SpoIIGA would confirm the localization of the proteins in the donor and acceptor cells. These observations may also verify whether SpoIIR is translocated or secreted during signaling.

Sporulation of *B. subtilis* involves additional intercellular signal transduction from the forespore to the mother cell. After completing engulfment, the late forespore-specific transcription factor σ^G^ is activated in the forespore (Tan and Ramamurthi 2014). The late mother cell-specific σ factor σ^K^ is produced in the mother cell as an inactive precursor, pro-σ^K^, and activated by the cleavage of the N-terminal pro-peptide by a metallo-intramembrane protease, SpoIVFB (Kroos and Akiyama 2013). Activation of SpoIVFB-mediated pro-σ^K^ processing requires a forespore signal, analogous to activation of SpoIIGA-mediated pro-σ^E^ processing by SpoIIR signaling. However, the molecular mechanisms of the two signaling pathways are distinct. SpoIVFB activity is initially inhibited by BofA and SpoIVFA embedded in the mother cell membrane (Cutting et al. 1990, Cutting et al. 1991a, Ramírez-Guadiana et al. 2018, Olenic et al. 2022). Inhibition is relieved by two proteases exported from the forespore, SpoIVB and CtpB (Pan et al. 2003, Cutting et al. 1991b, Pospíšil et al. 2020). SpoIVB cleaves the C-terminal extracellular domain of SpoIVFA, while CtpB cleaves the C-terminal extracellular domains of both BofA and SpoIVFA to trigger SpoIVFB-dependent pro-σ^K^ processing in the mother cell (Zhou and Kroos 2005, Campo and Rudner 2007). In this case, the signaling pathway involves secretion of proteases from the forespore into the intermembrane space between the forespore membrane and the mother cell membrane that has engulfed the forespore. Signaling involves proteolysis of inhibitory proteins. At this late stage of sporulation, peptidoglycan between the forespore and mother cell membranes is thinned during engulfment (Khanna et al. 2019), so signaling proteins exported from the forespore may access the surrounding mother cell membrane easily. In contrast, earlier during sporulation when the asymmetric septum forms, peptidoglycan and/or other factors may necessitate a mechanism to transfer the forespore signaling protein SpoIIR directly to the mother cell membrane (Fig. 1b).

The results in this study have shown that SpoIIR is capable to transduces the signal within the mother cell compartment instead to extracellular domain of SpoIIGA. Although direct evidence is lacking, one possible explanation for the observed signal transmission could involve transient membrane connections such as nanotube-like structures. Given that molecular transfer via such structures is facilitated between adjacent and sessile cells, this mechanism might be more feasible during sporulation than in our co-culture assays. Alternatively, localized membrane fusion events or other yet-unidentified contact-dependent processes might also mediate SpoIIR transfer. Further studies using high-resolution imaging will be required to clarify these possibilities.

## Supplementary Material

fnag055_Supplemental_Files
