# Supplementary figures and images for: Intercellular signal transduction within the mother cell compartment during Bacillus subtilis sporulation
Source: FEMS Microbiol Lett. 2026 May 5;373:fnag055. doi: 10.1093/femsle/fnag055 (PMC13174948; doi:10.1093/femsle/fnag055)

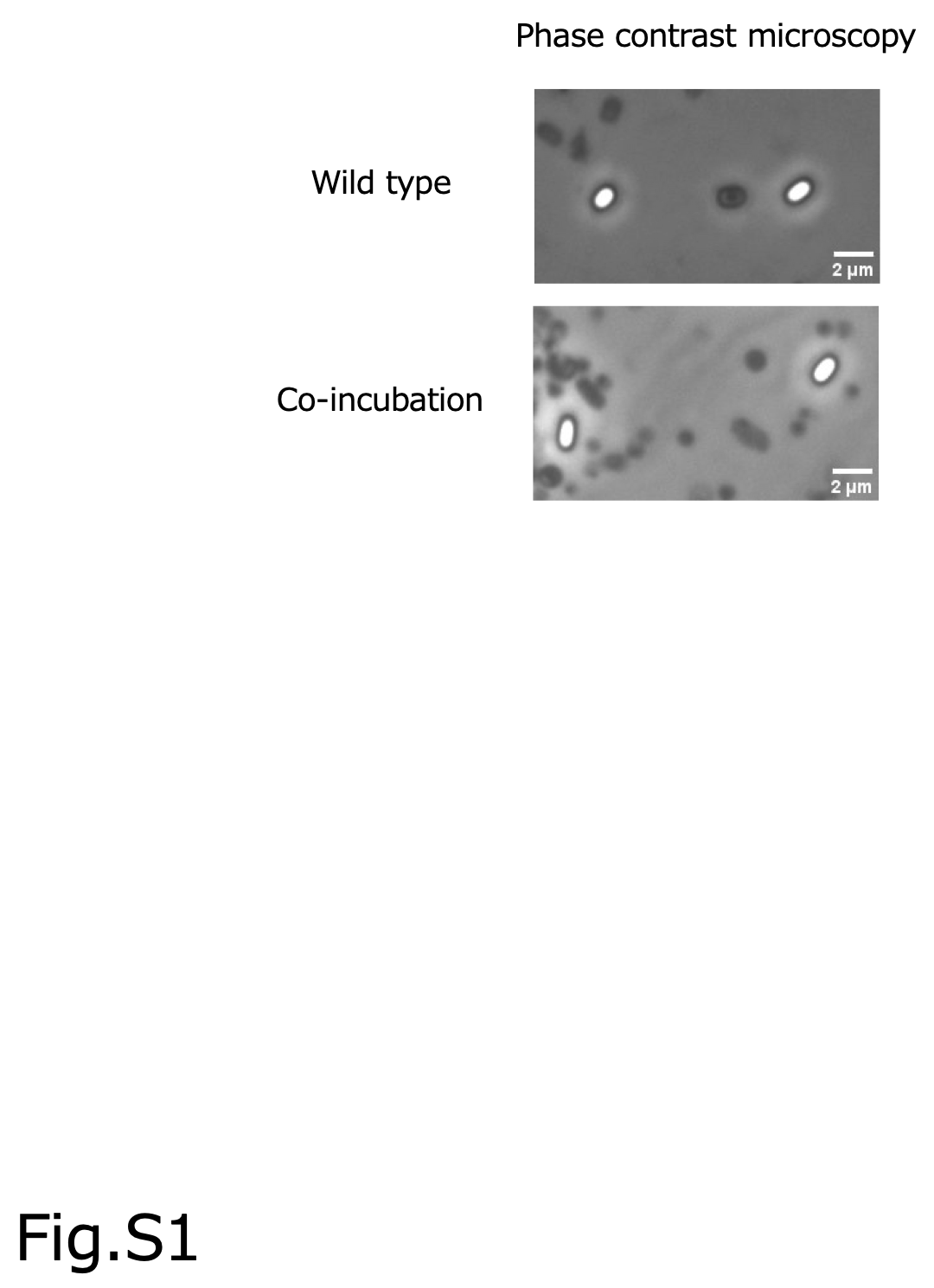

Supplement: fnag055_Supplemental_Files [file fnag055_supplemental_files.zip › Supplimental figures.tiff]
